# Faecal immunochemical testing for haemoglobin in detecting bowel polyps in symptomatic patients: multicentre prospective cohort study

**DOI:** 10.1093/bjsopen/zrac161

**Published:** 2023-03-08

**Authors:** Michael F Bath, Aman Malhi, Ruth M Ayling, Edward Seward, Kathy Pritchard-Jones, Helga E Laszlo, Allan Hackshaw, Michael R Machesney

**Affiliations:** Barts Health NHS Trust, The Royal London Hospital, London, UK; Cancer Research UK & UCL Cancer Trials Centre, University College London, London, UK; Barts Health NHS Trust, The Royal London Hospital, London, UK; North Central London Cancer Alliance, London, UK; University College London Hospitals NHS Foundation Trust, London, UK; North Central London Cancer Alliance, London, UK; University College London Hospitals NHS Foundation Trust, London, UK; UCL GOS Institute of Child Health, University College London, London, UK; North Central London Cancer Alliance, London, UK; Cancer Research UK & UCL Cancer Trials Centre, University College London, London, UK; Barts Health NHS Trust, The Royal London Hospital, London, UK; North East London Cancer Alliance, London, UK

## Abstract

**Background:**

Measurement of faecal haemoglobin using faecal immunochemistry testing is recommended in patients presenting with symptoms suspicious for colorectal cancer, to aid in triage and prioritization of definitive investigations. While its role in colorectal cancer has been extensively investigated, the ability of faecal immunochemistry testing to detect adenomas in symptomatic patients is unclear.

**Methods:**

A multicentre prospective observational study was conducted between April 2017 and March 2019, recruiting adults from 24 hospitals across England and 59 general practices in London who had been urgently referred with suspected colorectal cancer symptoms. Each patient provided a stool sample for faecal immunochemistry testing, in parallel with definitive investigation. A final diagnosis for each patient was recorded, including the presence, size, histology, and risk type of colonic polyps. The outcome of interest was the sensitivity of faecal immunochemistry testing in detecting the presence of adenomas.

**Results:**

Of 3496 patients included in the analysis, 553 (15.8 per cent) had polyps diagnosed. Sensitivity of faecal immunochemistry testing for polyp detection was low across all ranges; with a cut-off for faecal haemoglobin of 4 µg/g or lower, sensitivity was 34.9 per cent and 46.8 per cent for all polyp types and high-risk polyps respectively. The area under the receiver operating characteristic curve in detection probability was relatively low for both intermediate-risk (0.63) and high-risk polyps (0.63).

**Conclusion:**

While faecal immunochemistry testing may be useful in prioritizing investigations to diagnose colorectal cancer, if used as a sole test, the majority of polyps would be missed and the opportunity to prevent progression to colorectal cancer may be lost.

## Teaser

Faecal immunochemistry testing (FIT) is currently used in the UK to triage patients referred with symptoms suggestive of colorectal cancer; however, the role of FIT in the detection of adenomatous polyps, a known precursor to colorectal cancer, in symptomatic patients is unknown. Our study demonstrates that if FIT were used as a triage for further investigations in symptomatic patients, more than half of all high-risk adenomas would be missed, therefore current endoscopic investigations in symptomatic patients must remain to ensure that all adenomas can be identified and removed.

## Introduction

Colorectal cancer (CRC) is the fourth leading cause of cancer death in the world^1^. Despite ongoing improvements in its detection, around 52 000 individuals die every year in the USA from the disease^2^. With variability in its presentation, unfortunately many patients may not become symptomatic until an advanced stage^3^. Appropriate patient selection and early detection is therefore essential to improve survival rates, with effective CRC screening programmes being key to this^4^.

Faecal immunochemistry testing (FIT) has replaced faecal occult blood testing within many bowel cancer screening programmes worldwide in recent years^2,5^, due to benefits of greater sensitivity and ease of use^6,7^. Moreover, the role of FIT has further expanded and is now recommended by public bodies, such as the National Institute for Health and Care Excellence (NICE) in the UK, for use in patients presenting with low-risk symptoms of CRC^8^, to aid both in the referral from primary care and in the prioritization of definitive investigations^9,10^. FIT has been used less frequently in the USA, with colonoscopy remaining as the first line investigation, and this issue remains a subject of debate^11^.

Adenomatous polyps are a known precursor to CRC^12^ and the detection and removal of colonic polyps can prevent the development of CRC, in turn reducing the overall mortality from the disease^13^. While not all polyps need follow-up, high-risk adenomas (typically defined as five or fewer adenomas less than 10 mm, or three or fewer adenomas with at least one 10 mm or more) have a greater risk of developing CRC, therefore require follow-up surveillance colonoscopy^14^. Adenoma surveillance accounts for approximately 20 per cent of colonoscopies performed, placing ongoing pressures on some endoscopy services^15^.

While previous studies have suggested that a FIT result below a predefined threshold can rule out the diagnosis of CRC in individuals with symptoms suggestive of CRC^16^, a recent meta-analysis concluded that such use could lead to a CRC miss rate of up to 7.2 per cent^17^. Latest guidance from the Association of Coloproctology of Great Britain and Ireland and the British Society of Gastroenterology (BSG) has reinforced the view that FIT should continue to be used to aid in patient triage; however, there remains insufficient evidence at present in using FIT within a risk score with other clinical features^10^. The performance of FIT for adenoma detection in screening asymptomatic patients is known to be poor^18–20^, however the efficacy of FIT in adenoma detection for patients presenting with symptoms is less understood.

The aim of this analysis, using data collected as part of the quantitative FIT (qFIT) study, was to determine whether FIT can be used as a reliable test to identify the presence of adenomas in symptomatic patients.

## Methods

### Study design

A multicentre prospective observational study (the qFIT study) was run between April 2017 and March 2019, recruiting patients from 24 hospitals across England and 59 General Practices in London^17^. Primary and secondary care sites were invited through the National Institute for Health Research Clinical Research Network (NIHR CRN). National ethical approval was granted was granted by the UK NRES West Midlands—Solihull Research Ethics Committee (ref. 17/WM/0094) and the Health Research Authority (IRAS/213710), and the study was conducted following the STARD 2015 guideline for diagnostic accuracy studies^21^. Three patient representatives with previous experience with colorectal cancer were involved in the development of the patient information leaflet and the design of the FIT kit handout. This study is a subanalysis of a previously published paper on the role of FIT in ruling out CRC among patients presenting with ‘high-risk’ symptoms^17^.

Any adult (16 years or older) presenting to primary care with abdominal symptoms that merited an urgent referral for suspected CRC investigations were eligible^22^; symptoms in the urgent referral guidance were associated with a 3 per cent positive predictive value for CRC^22^. People who were under 16 years of age or were unable to understand instructions (including non-English speakers who did not have an interpreter) were excluded from the study. A FIT kit and a patient information booklet outlining the purpose of the research study were provided to patients by the primary care physician, hospital consultant, research nurse, or clinical nurse specialist.

The patient was asked to take a single sample from their next bowel movement (before completing any bowel preparation for subsequent colonoscopy or other examination) and post it to a central laboratory. By returning the FIT kit, the patient provided implied consent to participate in the study. Participation did not affect the patient’s clinical care. Participants were informed that the FIT result was for research purposes only (and they would not be informed of the result).

The FIT kit included a FIT sample collection device in a sealable plastic pouch (OC-Sensor™; Eiken Chemical Company, Tokyo, Japan) prelabelled with the patient’s name, National Health Service (NHS) number, a unique laboratory number, and a space to write the sample date; a copy of the urgent referral form or patient data sheet (containing information about the patient and the hospital where the examination took place); a patient experience survey consent form; and a prelabelled return envelope. The urgent referral form contained clinical data, such as symptoms, reasons for referral, medical history, and sociodemographic factors.

### Sample analysis

Samples for FIT were taken into an Eiken specimen collection device using the sampling probe in the lid and posted to the laboratory. The specimen collection devices were stored at 4°C until analysis, which took place within a week of receipt. F-Hb was measured by immunoturbidimetry using a single OC-Sensor (Eiken Chemical Co., Tokyo, Japan).

The coefficients of variation were 2.8 per cent at 14 µg/g and 3.0 per cent at 91 µg/g. External quality assurance was achieved via satisfactory performance in the relevant UK National External Quality Assessment Service schemes. The lower limit of quantification was 4 µg/g and the upper limit of the measuring range 200 µg/g. The laboratory is accredited by the UK Accreditation Service to ISO 15189 standards.

All test results were performed blinded to patient characteristics and outcomes. If a patient returned more than one sample, due to being given a test kit in both primary and secondary care during the same referral, or the patient had been investigated more than once, only the first test result was selected for inclusion in the analysis.

### Outcome definition

Clinical outcomes were collected for all patients who provided a viable sample, by requesting copies of examination reports from participating sites every month. All diagnoses were determined by reviewing endoscopy, radiology, and histology reports, clinic letters, and urgent referral forms provided by the participating sites. Patient and clinical data included symptoms, reasons for the referral, medical history, and sociodemographic factors. All diagnoses were verified by medical members of the central research team.

All neoplastic bowel polyps, either adenomatous polyps or sessile serrated polyps, were identified and were given a risk of either ‘low’, ‘intermediate’, or ‘high’ depending on their size and frequency; contemporary UK and European guidelines were used in this study^23,24^, with low risk defined as 1–2 adenomas less than 10 mm, intermediate risk as 3–4 small adenomas less than 10 mm or one adenoma 10 mm or more, and high risk as five or more adenomas less than 10 mm or three or more adenomas with at least one 10 mm or more.

Non-neoplastic polyps, such as hyperplastic, inflammatory, or pseudopolyps, were classified separately. Patients with a risk score for their polyps at first, second, or third examinations had their cumulative number and/or highest risk polyp taken as their final score. Remaining bowel pathology was classified as one of CRC, inflammatory bowel disease (colitis/proctitis), diverticulosis, haemorrhoids, normal examination, or procedure stopped/incomplete. Patients with concurrent polyps and CRC were classified as CRC and not included in the analysis, as our target group for this study was those without CRC in whom we could potentially identify polyps and plan for removal before they could progress to CRC.

### Outcome of interest

The outcome of interest was the sensitivity of FIT in detecting the presence of adenomas.

### Statistical analysis

Sensitivity (percentage of patients with adenomas whose FIT value exceeded a specified cut-off) and false-positive rates (or 100 minus specificity; percentage of patients without adenomas whose FIT value exceeded a specified cut-off) were calculated as measures of FIT test performance.

Receiver operating characteristic (ROC) curves were generated to illustrate the diagnostic ability of FIT, performed for all polyps and for each risk type respectively. Areas under the ROC curve were calculated to quantify FIT prediction performance. A multivariable logistic regression was performed for polyp detection outcome, including variables of patient age, patient ethnicity, patient sex, and f-Hb of 10 or higher.

Patients who were missing polypectomy information, and therefore polyp diagnosis, were not included in the denominator of any rates. Patients diagnosed with cancer following clinical investigations were not included in any of the analyses, however all other diagnoses were included.

## Results

### Study population

FIT kits were returned from 4676 patients in total, of which 3593 patients had both a viable sample for f-Hb measurement, valid polypectomy information, and a reported clinical diagnosis outcome following investigations (*Fig. S1*). Following exclusion of the 97 patients diagnosed with cancer (90 patients with CRC and seven patients with other cancer types), a final 3496 patients were included in our analyses.

The majority of patients (99 per cent) were recruited in secondary care. The median age was 67 years (69 per cent aged 60 years or older) and 53.5 per cent were female (*Table 1*). The prevalence of the five most reported clinical features recorded on the urgent referral form were change of bowel habit 1835 (52.5 per cent), rectal bleeding 970 (27.7 per cent), anaemia 684 (19.6 per cent), abdominal pain 427 (12.2 per cent), and weight loss 312 (8.9 per cent). The first investigation was colonoscopy (77.7 per cent), CT colon (14.5 per cent), and flexible sigmoidoscopy (7.3 per cent), with 78.0 per cent having a colonoscopy at any time point.

**Table 1:** Baseline characteristics of study patients

Characteristics	Polypectomy outcome		
	Total *n* = 3496	Any polyp *n* = 553	No polyp *n* = 2943
**Age (years), median (i.q.r.)**	67 (57–75)	68 (59–74)	67 (56–75)
**Age group (years)** < 30	21 (0.6)	0 (0)	21 (0.7)
30–39	76 (2.2)	8 (1.5)	68 (2.3)
40–49	260 (7.4)	33 (6.0)	227 (7.7)
50–59	726 (20.8)	105 (19.0)	621 (21.1)
60–69	955 (27.3)	174 (31.4)	781 (26.5)
70–79	974 (27.9)	174 (31.4)	800 (27.2)
80–89	462 (13.2)	59 (10.7)	403 (13.7)
90+	22 (0.6)	0 (0)	22 (0.8)
**Sex ratio (M:F)**	1616 (46.2):1871 (53.5)	321 (58.1):232 (42.0)	1295 (44.0):1639 (55.7)
Missing data	9 (0.3)	0 (0)	9 (0.3)
**Ethnicity**			
Black/Black British	157 (4.5)	18 (3.3)	139 (4.7)
Asian/Asian British	219 (6.3)	39 (7.1)	180 (6.1)
Other Asian*	70 (2.0)	12 (2.2)	58 (2.0)
White	820 (23.5)	134 (24.2)	686 (23.3)
British mixed	626 (17.9)	105 (19.0)	521 (17.7)
Multiple/other	197 (5.6)	30 (5.4)	167 (5.7)
Missing data	1407 (40.3)	215 (38.9)	1192 (40.5)

Values are *n* (%) unless otherwise indicated. *The ethnicity of ‘Other Asian’ consisted of those with Chinese ethnicity or Asian ethnicity other than Indian/Indian British, Pakistani/Pakistan British, or Bangladeshi/Bangladeshi British. i.q.r., interquartile range.

Neoplastic polyps alone (adenomatous or sessile serrated polyps, without a concurrent cancer diagnosis) were identified in 553 patients (15.8 per cent). Other diagnoses in those without cancer included diverticulosis in 1101 patients (31.5 per cent), haemorrhoids in 526 patients (15 per cent), colitis in 286 patients (8.2 per cent), and other polyp types in 250 patients (7.0 per cent).

Of note, in this cohort, 464 patients had undergone a polypectomy procedure but their cancer status was not reported to the research team. Among this subset, only two were found to have low-risk polyps and the remaining 462 (99.6 per cent) had no polyps detected. The baseline characteristics of the study sample with and without this patient subset were very similar (*Table S1*); including these patients did not materially affect the FIT test performance for detecting polyps (*Table S2*).

### Detection of polyps

Neoplastic polyps alone (adenomatous or sessile serrated polyps, without a concurrent cancer diagnosis) were identified in 553 patients (15.8 per cent). Of the 553 polyps that were diagnosed, 62.8 per cent were classified as low risk, 28.8 per cent as intermediate risk, and 8.5 per cent as high risk.

At the lowest f-Hb cut-off (4 µg/g or higher), FIT could only detect 34.9 per cent (95 per cent c.i. 30.9 to 39.0) of patients diagnosed with any polyp at investigation (low, intermediate, or high risk), with a false-positive rate of 25.5 per cent (95 per cent c.i. 23.9 to 27.1) (*Table 2*). At the highest f-Hb cut-off (200 µg/g or higher), sensitivity was 6.0 per cent (95 per cent c.i. 4.1 to 8.3) and the false-positive rate was 4.1 per cent (95 per cent c.i. 3.4 to 4.8) respectively.

**Table 2:** Test performance of faecal immunochemistry testing for neoplastic polyps (low, intermediate, high risk), at different faecal haemoglobin cut-offs

F-Hb level, µg/g	Sensitivity (true positives)				False-positive rate (false positives) *n* = 2943
	All polyps *n* = 553	Low-risk polyps *n* = 347	Intermediate-risk polyps *n* = 159	High-risk polyps *n* = 47	
≥4	34.9 (193)	26.8 (93)	49.1 (78)	46.8 (22)	25.5 (750)
≥6	31.6 (175)	23.1 (80)	47.2 (75)	42.6 (20)	22.5 (662)
≥10	26.8 (148)	17.3 (60)	42.8 (68)	42.6 (20)	18.6 (548)
≥20	20.3 (112)	11.5 (40)	33.3 (53)	40.4 (19)	13.4 (394)
≥50	11.9 (66)	6.6 (23)	19.5 (31)	25.5 (12)	7.7 (228)
≥80	9.6 (53)	5.5 (19)	17.0 (27)	14.9 (7)	6.2 (181)
≥100	8.1 (45)	5.2 (18)	14.5 (23)	8.5 (4)	5.4 (160)
≥120	6.9 (38)	4.3 (15)	12.0 (19)	8.5 (4)	5.0 (146)
≥150	6.3 (35)	3.8 (13)	11.3 (18)	8.5 (4)	4.5 (133)
≥200	6.0 (33)	3.5 (12)	10.7 (17)	8.5 (4)	4.0 (119)

Values are % (*n*). F-Hb, faecal haemoglobin.

For high-risk polyps only, the lowest f-Hb cut-off (4 µg/g or higher) had test sensitivity of 46.8 per cent (95 per cent c.i. 32.1 to 61.9) and false-positive rate of 26.7 per cent (95 per cent c.i. 25.2 to 28.2) (*Table 2*). This indicates that FIT could miss more than half the cases of high-risk polyps in this symptomatic patient population if used alone. For high- or intermediate-risk polyps combined, at the lowest f-Hb cut-off (4 µg/g or higher), test sensitivity levels were 48.5 per cent (95 per cent c.i. 41.5 or 55.6) and the false-positive rate was 25.6 per cent (95 per cent c.i. 24.1 to 27.1).

**Fig. 1:**
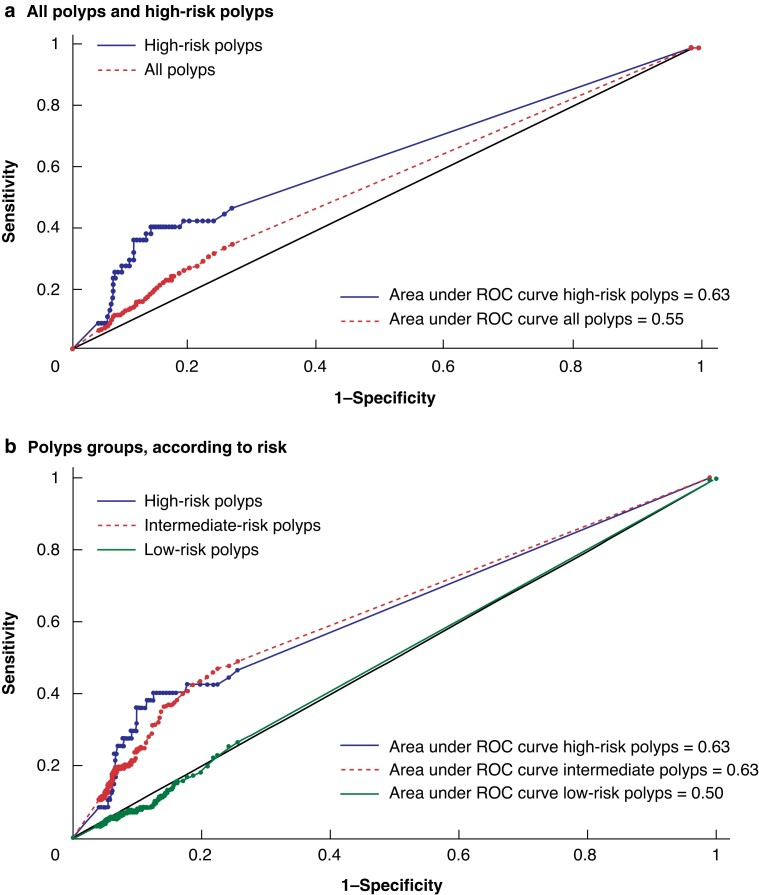
**ROC curve** **a** All polyps and high-risk polyps; **b** Polyps groups, according to risk. ROC, Receiver operating characteristic.

The area under the ROC curve was 0.50 for low-risk polyps (no better than chance alone), and only modest for intermediate-risk (0.63) and high-risk (0.63) polyps (*Fig. 1*). Multivariate logistic regression showed that a f-Hb higher than 10 µg/g was associated with a diagnosis of all polyps (OR 1.40, 95 per cent c.i. 1.06 to 1.85; *P* = 0.018) and for high-risk polyps (OR 3.10, 95 per cent c.i. 1.49 to 6.46; *P* = 0.002) (*Table S3*).

## Discussion

FIT is used both within CRC screening programmes and to aid in the prioritization of investigations in patients presenting with abdominal symptoms. The detection and management of adenomas, especially high-risk adenomas, is an essential part of this symptomatic colorectal diagnostic pathway to prevent the progression to CRC. This work has demonstrated that, at any cut-off for f-Hb, the sensitivity rates for detection of polyps with FIT are unacceptably low. At current standard thresholds, FIT will miss around two-thirds of all polyps and more than half of high-risk polyps.

It has previously been demonstrated that f-Hb assessed by FIT is strongly correlated to CRC risk, with recent meta-analyses reporting the sensitivity of FIT for CRC in symptomatic patients between 92 per cent and 94 per cent^25,26^, therefore can be useful in the triage of referred patients with abdominal symptoms^16,27^. However, the FIT ‘miss rate’ for CRC remains at approximately 7 per cent^17^ and therefore colonoscopy still remains the recommended standard for the diagnosis of CRC^28^. This work shows the same holds true for polyp detection; FIT is known to be a poor prediction of polyp presence in screening patients^18–20^ and this present data has added to this body of evidence, demonstrating similarly poor prediction of polyp presence in patients presenting with symptoms indicating a risk of CRC.

For early diagnosis and prevention of CRC, it remains essential for adenomas to be identified and removed, and higher-risk cases to be followed up with surveillance colonoscopy^13,17,29^. A study published in 2019 showed that the post-colonoscopy CRC rate at 3 years is between 3.6–7.4 per cent^30^, implying that these cancers have developed since the original endoscopic examination, progressing as part of the adenoma sequence, thereby reinforcing the need for diligent early adenoma detection and removal. Indeed, the Bowel Scope programme in England, where a flexible sigmoidoscopy was offered to individuals turning 55 years old, has shown that identification and removal of very small polyps bestows a survival benefit lasting up to 17 years^31^. The adenoma detection rate reported in this current study of 16 per cent is in keeping with the published literature from other similar cohorts^18,19,32^.

The present research has shown that if FIT were to be used alone, around two-thirds of all adenomas and half of high-risk adenomas would be missed. Current NICE guidance recommends an f-Hb cut-off of 10 µg/g^8^; however, at such a level, for every 1000 symptomatic patients tested, based on the authors' data, then 158 patients would have a polyp and 116 of these would be missed, and 13 patients would have a high-risk adenoma of which eight would be missed. While multiple pathways are available for patients to access endoscopic assessment, a sizeable proportion of patients come through the suspected cancer pathways, where FIT is most utilized^10^; with FIT showing poor prediction of polyp presence, current use of endoscopic assessment to ensure adequate identification and removal is essential.

The use of FIT has likely diverted more patients onto more CRC-focused pathways, however presently there is no consensus for the use of FIT in detecting adenomas in these patient groups. Use of novel biomarkers, such as urinary volatile organic compounds, to help improve FIT sensitivity remains in its infancy^33^ and whether such adjuncts with FIT would change adenoma detection remains to be determined. At present the role of FIT in the follow-up of patients who have undergone recent polyp removal is controversial, as current miss rates for CRC and advanced adenomas at 3 years post-endoscopy are 30–40 per cent and 40–70 per cent respectively^34^. As such, until improvements are made to present methods, endoscopic assessment for polyp identification and removal must remain the mainstay.

This study has a few limitations. First of all, it included patients referred with abdominal symptoms indicating a 3 per cent risk of CRC, therefore the identification of polyps was opportunistic rather than a screening method, however the role of FIT in screening patients has already been extensively examined^18–20,35^. Despite a large number of participants, although the total number of polyps detected were in the expected range, the absolute number of high-risk polyps identified was low, making the study susceptible to type II error. The selection of definitive investigation in the study was part of the pragmatic real-life clinical practice at the participating sites, not under influence by the investigators. Not all patients underwent endoscopic investigation either, 14.4 per cent had CT virtual colonoscopy, where smaller polyps may not have been identified and accounted for; for those that had endoscopic investigation, some patients only had a flexible sigmoidoscopy, therefore more proximal polyps may also have been missed. Due to the timing of data collection, the definition of high-risk polyps is based on previous guidelines, and does not reflect the more recently published BSG guidelines^14^.

Polyps are a precursor of CRC and their early detection and ablation is an essential intervention in the prevention of CRC, especially for people that are at high risk. FIT may be useful in stratifying the timing of investigations for patients presenting with symptoms that may be caused by CRC in resource-limited health economies; however, if FIT were to replace current investigative pathways based on symptoms indicating definitive investigation, nearly three-quarters of all adenomas, including more than half the high-risk polyps, in symptomatic patients would be missed. To ensure adenoma detection rates remain high, endoscopic assessment must not be reduced from current standards of practice.

## Supplementary Material

zrac161_Supplementary_Data

## Data Availability

The authors confirm that the data supporting the results in the paper will be accessible upon request from the corresponding author.
