# Supplementary material for: Faecal immunochemical testing for haemoglobin in detecting bowel polyps in symptomatic patients: multicentre prospective cohort study
Source: BJS Open. 2023 Mar 8;7(2):zrac161. doi: 10.1093/bjsopen/zrac161 (PMC9994599; doi:10.1093/bjsopen/zrac161)

**Faecal Immunochemical Testing for Haemoglobin is Unreliable for Detecting Bowel Polyps in Symptomatic Patients: Multi-Centre Prospective Cohort Study**

Michael F. Bath^1^, Aman Malhi^2^, Ruth M. Ayling^1^, Edward Seward^3,4^, Kathy Pritchard-Jones^3,4,5^, Helga E Laszlo^3^, Allan Hackshaw^2^, Michael R. Machesney^1,6^

*^1^ Barts Health NHS Trust, The Royal London Hospital, Whitechapel Road, London E1 1BB, UK*

*^2^ Cancer Research UK & UCL Cancer Trials Centre, University College London, 90 Tottenham Court Road, London W1T 4TJ, UK*

*^3^ North Central London Cancer Alliance, 47 Wimpole Street, London W1G 8SE, UK*

*^4^ University College London Hospitals NHS Foundation Trust, 235 Euston Road, London NW1 2BU, UK*

*^5^ UCL GOS Institute of Child Health, University College London, 30 Guilford Street, London WC1N 1EH, UK*

*^6^ North East London Cancer Alliance, Unex Tower, 5 Station Street, London E15 1DA, UK*

**Corresponding Author**Mr. Michael R. Machesney, Department of Surgery, Whipps Cross University Hospital, Barts Health NHS Trust, London, UK; [michael.machesney@nhs.net](mailto:michael.machesney@nhs.net)

ORCHID ID: 0000-0002-8088-6766

| **Supplementary Figures and Tables** |  |
| --- | --- |
| Table S1 | *Page 2* |
| Table S2 | *Page 3* |
| Table S3 | *Page 4* |
| Figure S1 | *Page 5* |

*Table S1 - Baseline characteristics of patients with a valid FIT test result, polypectomy information and no diagnosis of cancer (including 464 patients with a missing cancer status)*

| **Characteristics** | **Polypectomy Outcome** | | |
| --- | --- | --- | --- |
|  | Total  N = 3960 (%^1^) | Any Polyp  N = 555 (%^2^) | No Polyps  N = 3405 (%^2^) |
| *Median Age, years (range)* | 67 (17-100) | 68 (31-89) | 67 (17-100) |
| *Age group, years*  <30 | 25 (0.6) | 0 (0)  8 (1.4) | 25 (0.7)  76 (2.2) |
| 30-39  40-49 | 84 (2.1) |  |  |
|  | 298 (7.5) | 33 (6.0) | 265 (7.8) |
| 50-59 | 801 (20.2) | 106 (19.1) | 695 (20.4) |
| 60-69 | 1046 (26.4) | 175 (31.5) | 871 (25.6) |
| 70-79 | 1100 (27.8) | 174 (31.4) | 926 (27.2) |
| 80-89 | 563 (14.2) | 59 (10.6) | 504 (14.8) |
| 90+ | 30 (0.8) | 0 (0) | 30 (0.9) |
| Missing data | 13 (0.3) | N/A | 13 (0.4) |
| *Gender, number*  Female | 2078 (52.5) | 233 (42.0) | 1845 (54.2) |
| Male | 1845 (46.6) | 322 (58.0) | 1523 (44.7) |
| Missing data | 37 (0.9) | N/A | 37 (1.1) |
| *Ethnicity, number*  Black/Black British | 175 (4.4) | 19 (3.4) | 156 (4.6) |
| Asian/Asian British | 250 (6.3) | 40 (7.2) | 210 (6.2) |
| Other Asian^3^ | 80 (2.0) | 12 (2.2) | 68 (2.0) |
| White | 935 (23.6) | 134 (24.1) | 801 (23.5) |
| British Mixed | 698 (17.6) | 105 (18.9) | 593 (17.4) |
| Multiple/Other | 216 (5.5) | 30 (5.4) | 186 (5.5) |
| Missing data | 1606 (40.6) | 215 (38.7) | 1391 (40.9) |

*^1^ Column percentages*

*^2^ Row percentages to show proportion of patients with/without polyps within each demographic subgroup*

*^3^The ethnicity of ‘Other Asian’ consisted of those with Chinese ethnicity or Asian ethnicity other than Indian/Indian British, Pakistani/Pakistan British, or Bangladeshi/Bangladeshi British*

*Table S2 - Test performance of FIT for neoplastic polyps (low, intermediate, high-risk) at different f-Hb cut offs, including 464 patients for whom we did not know their cancer outcome status*

| *F-Hb Level, µg/g* | Sensitivity %  (True Positives, n) | | | | False-Positive Rate, %  (False positives, n)  n=3405 |
| --- | --- | --- | --- | --- | --- |
|  | All Polyps  n=555 | Low-Risk Polyps  n=349 | Intermediate-Risk Polyps  n=159 | High-Risk Polyps  n=47 |  |
| *≥4* | 34.8 (193) | 26.7 (93) | 49.1 (78) | 46.8 (22) | 26.3 (895) |
| *≥6* | 31.5 (175) | 22.9 (80) | 47.2 (75) | 42.6 (20) | 23.2 (790) |
| *≥10* | 26.7 (148) | 17.2 (60) | 42.8 (68) | 42.6 (20) | 19.2 (653) |
| *≥20* | 20.2 (112) | 11.5 (40) | 33.3 (53) | 40.4 (19) | 13.7 (467) |
| *≥50* | 11.9 (66) | 6.6 (23) | 19.5 (31) | 25.5 (12) | 7.98 (268) |
| *≥80* | 9.5 (53) | 5.4 (19) | 17.0 (27) | 14.9 (7) | 6.1 (207) |
| *≥100* | 8.1 (45) | 5.2 (18) | 14.5 (23) | 8.5 (4) | 5.3 (182) |
| *≥120* | 6.8 (38) | 4.3 (15) | 12.0 (19) | 8.5 (4) | 4.9 (167) |
| *≥150* | 6.3 (35) | 3.7 (13) | 11.3 (18) | 8.5 (4) | 4.5 (153) |
| *≥200* | 5.9 (33) | 3.4 (12) | 10.7 (17) | 8.5 (4) | 4.1 (138) |

*Table S3 - Multivariable logistic regression for the predictors of polyps, at a f-Hb cut off of 10 µg/g*

|  | | **All polyps, n=2089** | | **High-risk polyps, n=1782** | |
| --- | --- | --- | --- | --- | --- |
| *Variable* | | Odds Ratio (95% CI) | P-Value | Odds ratio (95% CI) | P-Value |
| *f-Hb (µg/g)* | *≥10* | 1.40 (1.06-1.85) | 0.02 | 3.10 (1.49-6.46) | 0.002 |
|  | *<10* | Reference | NA | Reference | NA |
| *Age* | | 1.01 (1.00-1.02) | 0.21 | 1.02 (0.99-1.05) | 0.21 |
| *Ethnicity* | *Black/Black British* | 0.69 (0.40-1.17) | 0.17 | 0.93 (0.27-3.25) | 0.91 |
|  | *Asian/Asian British* | 1.12 (0.75-1.66) | 0.58 | 0.24 (0.03-1.81) | 0.17 |
|  | *Other Asian* | 1.02 (0.53-1.96) | 0.95 | 0.67 (0.09-5.15) | 0.70 |
|  | *British Mixed* | 1.02 (0.77-1.35) | 0.89 | 0.47 (0.20-1.15) | 0.10 |
|  | *Multiple/Other* | 0.92 (0.60-1.42) | 0.72 | 0.23 (0.03-1.72) | 0.15 |
|  | *White* | Reference | NA | Reference | NA |
| *Gender* | *Male* | 1.73 (1.36-2.19) | <0.001 | 1.45 (0.71-2.99) | 0.31 |
|  | *Female* | Reference | NA | Reference | NA |

*Figure S1 - qFIT Polyp Study Flow Diagram*


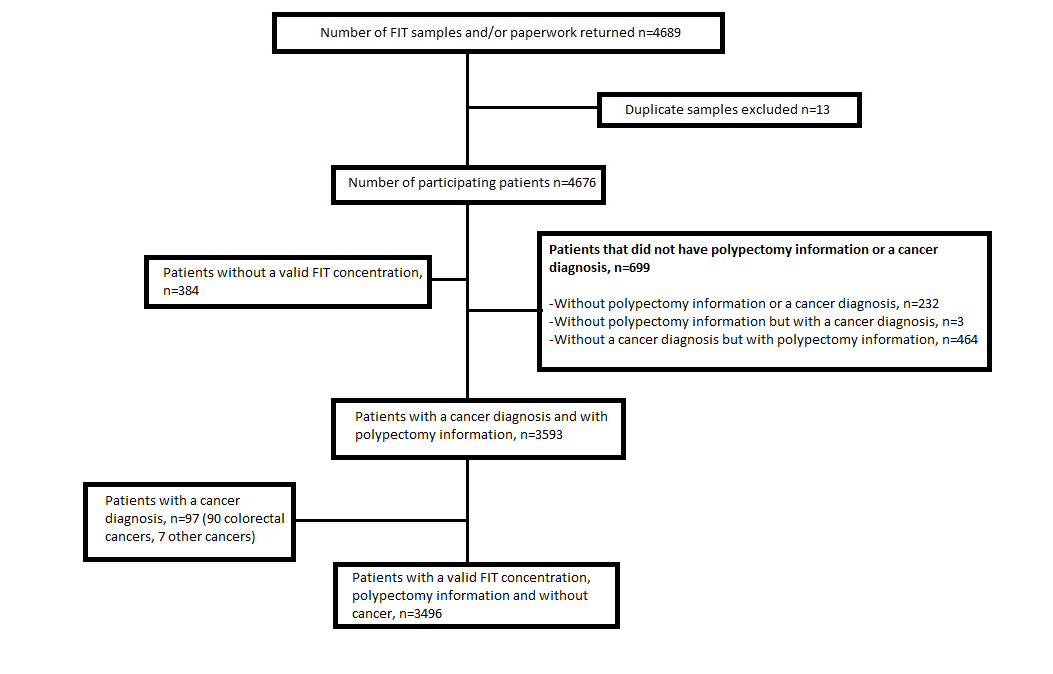

Supplement: zrac161_Supplementary_Data [file zrac161_supplementary_data.docx]
